# Conductive silicone elastomers electrodes processable by screen printing

**DOI:** 10.1038/s41598-019-49939-8

**Published:** 2019-09-16

**Authors:** Jose Enrico Q. Quinsaat, Iurii Burda, Ronny Krämer, Daniel Häfliger, Frank A. Nüesch, Mihaela Dascalu, Dorina M. Opris

**Affiliations:** 1 Swiss Federal Laboratories for Materials Science and Technology Empa, Laboratory for Functional Polymers, Ueberlandstr. 129, CH-8600 Dübendorf, Switzerland; 2 Swiss Federal Laboratories for Materials Science and Technology Empa, Laboratory for Mechanical Systems Engineering, Ueberlandstr. 129, CH-8600 Dübendorf, Switzerland; 3 Sateco AG, Tumigerstr. 111, CH-8606 Naenikon-Uster, Switzerland; 4 Ecole Polytechnique Fédérale de Lausanne (EPFL), Institut des Matériaux, Station 12, CH 1015 Lausanne, Switzerland

**Keywords:** Materials chemistry, Actuators, Sensors and biosensors

## Abstract

Conductive inks consisting of graphene and carbon black conductive fillers into a polydimethylsiloxane (PDMS) matrix, which can be processed into thin films by screen printing are developed. The influence of filler composition and content on mechanical and electrical properties of the conductive composites is investigated. The best composites were evaluated as electrode material for dielectric elastomer actuators and for piezoelectric sensors. With increasing filler content, the electrical properties of the resulting composites of graphite nanoplates (GNPs) or a binary mixture of GNPs and carbon black (CB) with PDMS (M_w_ = 139 kg/mol) are enhanced. Hence, PDMS composites filled with GNPs (42 wt.%) or a binary mixture of GNPs/CB (300/150 ratio, 30 wt.% of total filler loading) exhibited constant contact resistance values of 0.5 and 5 Ω determined in life-cycle test, respectively, thus rendering them suitable as electrode materials for piezosensors. On the other hand, dielectric elastomer actuators require more flexible electrode materials, which could be tuned by varying the polymer molecular weight and by reducing the filler content. Therefore, a composite consisting of PDMS (M_w_ = 692 kg/mol) and a binary filler mixture of GNPs/CB (150/75 ratio, 18 wt.% of total filler loading) was used for producing the electrodes of dielectric elastomer transducers (DETs). The produced DETs with different electrode thicknesses were characterized in terms of their performance. The negligible hysteresis of the electrode materials is favorable for sensor and actuator applications.

## Introduction

The tremendous development of elastic piezoelectric sensors and dielectric elastomer transducers (DET) in the last decade stimulated the search for conductive stretchable electrode materials by the research community worldwide. Both, the piezoelectric sensors as well as the DET are elastic capacitors composed of a thin elastic film coated with two electrodes. They require elastic conductive electrodes which have a low hysteresis after many operation cycles. Such elastic electrodes can be prepared either by direct printing of a conductive ink on an elastic substrate or by using a composite approach, in which conductive fillers are blended into an elastic matrix, which ensures elasticity^1,2^. The most used dielectric elastomer for both piezoelectric sensors as well as for DET is polydimethylsiloxane (PDMS).

Previous attempts to deposit conductive materials directly on a PDMS surface, although successful, required an additional surface treatment due to the low surface energy of PDMS, which leads to poor adhesion of electrode material to the polymer film surface^3–6^. Often, such surface treatments alter the chemical composition as well as the mechanical properties of the dielectric used. Therefore, this approach is considered inconvenient for the large scale industrial production of devices. Carbon nanotubes (CNTs) have attractive conductivity and inks containing them have been used to fabricate electrodes by for example spray coating, screen printing, and inkjet printing. While the resulting properties are promising, concerns regarding the safe use of CNT may hinder their industrial implementation^7–9^. Ji *et al*. used a combination of multiwalled CNTs and poly(alkylthiophene) to form thin layers at the air-water interface. Such layers were transferred onto a PDMS membrane using the Langmuir-Schaefer technology. Unfortunately, large scale production of such electrodes is still challenging^10^. A screen printable graphene ink with excellent conductivity and flexibility was developed by Hyun *et al*., however no information about how conductivity is changing with strain has been given^11^.

To avoid delamination between the electrodes and the dielectric during operation, a very good adhesion between them is needed. Electrodes consisting of conductive particle fillers within the PDMS matrix are attractive, since they can be applied directly on the PDMS dielectric layer without any surface treatment. Additionally, the mechanical properties of such composites can be easily tailored by parameters such as the molecular weight and the cross-linking density^12,13^. To ensure the conductivity of PDMS composites, silver nanoparticles (AgNPs)^14^, silver nanowires (AgNWs)^15^, polyaniline (PANI)^16^, carbon black (CB)^17^, CNTs^18^, carbon nanofibers (CNF)^19^ or graphite/graphene^20^ have been used as fillers. Composites of MWCNT in PDMS with conductivity up to 0.01 S/cm were also reported, but the functionality of these electrode materials in real devices was not evaluated^21^. Carbonaceous fillers such as graphite or graphene are currently highly sought because they are commercially available, cheap, lightweight, and highly conductive. Such composites may find application as electrode materials in dielectric elastomer transducers or in piezoresistive or piezoelectric sensors^22–24^. Parviz *et al*. reported the preparation of graphene/PDMS nanocomposites exhibiting a conductivity of 2.2 S/cm at a graphene content of 1.7 vol. %, which is quite attractive. However, no information regarding how the electrical properties change with strain was given^25^. Cazacu *et al*. reported a 20 wt% CB/PDMS composite with a moderate conductivity of 0.1 S/cm^26^. Opris *et a*l. reported the preparation of PDMS/graphite nanocomposites with a conductivity of 0.35 S/cm with graphene nanoplates (GNPs) content of 20 wt.%, while the conductivity below this filler content was unsatisfactory. This composite was used as electrode in actuators, where it showed good compliancy and conductivity when stretched up to 37%. However, a higher conductivity is desired for piezoelectric sensors, especially when used at high frequencies^20^. Commercial elastic composites consisting of CB/PDMS were screen printed by Fasolt *et al*. Although this material works well in actuators, its conductivity was found to be too low for the piezosensor applications^27^.

The conductivity of carbonaceous composites can be further enhanced through the combination of various filler materials. Previous reports show that the mixture of silver nanoparticles/graphene^15^, silver nanoparticles/CNTs^28^, graphite/CB^29^, graphite/MWCNTs^30^, or CB/CNTs^31–33^ led to composites with high conductivities which is attributed to the combination of large and small structures beneficial for creating a large conductive network within the composite. Despite the abundant literature on conductive elastomers, only very few reports exist in which the developed materials were tested in real devices and where their lifetime is evaluated.

It was the aim of this work to prepare conductive composites that can be processed by screen printing and to evaluate their mechanical, electrical properties and lifetime in real devices. For this, two cheap and commercially available conductive fillers, specifically CB and GNPs, were selected. Composites with different concentrations and proportions of the two fillers in elastic PDMS matrices were prepared. The resulting composites were lightweight, cheap, and non-hazardous to health. With the best material developed, electrodes for piezoelectric sensors as well as electrodes for DETs were screen printed. The performance of such electrodes was evaluated^34^.

## Experimental Part

### Materials

#### Experimental materials

Graphite nanoplatelets (GNP, xGNP-M-25, 6–8 nm × 25 µm sheets) were purchased from XG Sciences Inc., USA and carbon black (Ketjenblack EC-600 JD, primary particle size 34 nm) from Akzonobel. Neukasil A7 was purchased from Altropol Kunststoffe, Neukasil RTV-23 from Swiss Composite, titanium-2-ethylhexoxide, dibutyltindilaurate (DBTDL) and ethyl triacetoxysilane (ETAS) from Alfa Aesar. Linear hydroxyl end-functionalized polydimethylsiloxane PDMS *M*_w_ = 139 kg/mol (PDMS 139 k) was purchased from ABCR, while PDMS *M*_w_ = 692 kg/mol (PDMS 692 k) was prepared according to the literature^35^.

#### PDMS substrate

The PDMS substrate was prepared by mixing Neukasil RTV-2 and Neukasil A7 at a mass ratio of 10 to 3 in toluene at a concentration of 0.78 g/mL prior to casting on a Teflon substrate by doctor blading. The thickness of the substrate was 600–700 µm.

#### PDMS composites

The filler component(s) were dispersed in toluene (~10 mL) by tip sonication for 2 minutes, mixed with PDMS (750 mg) and ETAS (187 µL) in the three-roll-mill (Exakt, type 80 S) for 10 minutes. After the second insertion path, the composite was redispersed in toluene, stirred at room temperature for ~2 h, then treated with a 50% of titanium-2-ethylhexoxide in toluene (187 µL), bath sonicated for 5 min, followed by casting on the PDMS substrate by doctor blading. The thickness of the composites was 100–200 µm. The composites were let to stand at room temperature over a few days prior investigating their properties.

#### Characterization methods

SEM images were obtained with a Nova NanoSEM 230 FEI. TGA was conducted with a Perkin Elmer TGA7 at a heating rate of 20 °C min^−1^ under a He gas flow. Tensile tests were performed with a Zwick Z010 tensile test machine. The Young’s modulus was determined from the slope of the stress–strain curves at ≤10% strain. The conductivities were calculated from sheet resistance measurement with a four point set up based on the van der Pauw method.

#### Electromechanical characterization

For the application as the electrode material in both piezoelectric sensors as well as DETs, it is essential to evaluate the PDMS composites in terms of their electromechanical behavior, that is, the response of their electrical resistance on the imposed external deformation. For this purpose, the dumb-bell samples with the gauge length of 10 mm and width of 10 mm were cut and axially strained using testing facility Zwick/Roell. This testing equipment consists of linear drives and video-extensometer for precise strain measurements. For the testing (see Fig. S1) the sample was fastened by clamps with the electrode facing downwards. Special markers were placed on the top of the sample for the measurements of deformation. The straining of the samples was performed with the displacement rate of 4 mm/min in steps with soaking periods of 1 min between the steps, during which the facility was kept at constant strain. During the soaking period the measurements of electrical resistance according to four-point probe method were performed. The direct current (I = 4.5 mA) was conducted through the sample via the clamps, and the measurement of the voltage was performed with a multimeter by applying the voltage measurement pins to the surface of the electrode. After measurement the voltage pins were disengaged form the electrode until next strain level was reached. The electrical resistance was then calculated according to Ohms law.

For the sheet conductivity, four-point measurement was used with a Jandel RM3-AR instrument. The conductivity was then calculated according to: σ = 1/(ρ_ϒ_ × d), where *σ* is the conductivity, *ρ*_ϒ_ is the sheet resistivity, and *d* is the thickness of the composite. The contact resistance Rc was measured by placing the conductive films on a PCB (Fig. S2c) and then adding 50 g circular cylindrical weight with a diameter of 21 mm on top of the film using a multimeter 1Z43.

The long-term stability of the conductive materials was evaluated by measuring the contact resistance Rc by preparing 3 mm pills from the conductive films, placing and gluing it on a PCB (Fig. S2c) and then the pills were subjected to press/release cycles in order to monitor the their stability with continuous operation up to 100000 cycles.

#### Screen printing

The screen printing was conducted with a screen from Serilith AG with a mesh of 24–140 W (140 μm), 105 cm^3^/m^2^, 26 × 36 cm, diameter 11 mm.

#### Screen printing of the electrode for actuators

The composite used as electrode for actuators was printed on an Elastosil film 50 μm thick. For this a solution of GNPs/CB 300/150 (18 wt.%) PDMS 692 k composite (1 g) in toluene (8.3 mL) was used (Table 1, Entry 10). To achieve electrodes of 16.5 μm and 25.5 μm thick, the composite was printed once or twice, respectively. To achieve electrodes with a thickness of 7.5 μm, a solution of GNPs/CB 300/150 (18 wt.%) PDMS 692 k composite (1 g) in toluene (11.7 mL) was used. The diameter of the printed electrodes was 11 mm. After the electrodes were cross-linked, the actuators were biaxially prestrained by 7.5%. A FUG HCL-35–12500 high voltage source served as power supply for actuator tests. The voltage was increased by 100 V steps every 2 s up to breakdown. The actuation strain was measured optically as the extension of the diameter of the electrode area *via* a digital camera, using an edge detection tool of a LabView program to detect the boundary between the black electrode area and the transparent silicone film.

**Table 1:** List of prepared composites using GNPs and CB as conductive fillers.

	*M*_w_(PDMS) [kg∙mol^−1^]^a)^	GNPs [mg]	CB [mg]	Total filler [wt.%]	Volume conductivity σ [S/cm]	Contact resistance R_c_ [Ω]^b)^
1	139	300	200	32	5.1 ± 0.6	1.2
2	139	300	150	30	7.8 ± 1.3	1.7
2b	692	300	150	30	6.2 ± 1.2	1.4
3	139	300	70	26	4.8 ± 0.9	2
4	139	300	40	24	2.2 ± 0.7	1.9
5	139	750	—	42	1.4 ± 0.6	1.4
6	692	450	—	30	0.9 ± 0.2	2.4
7	139	450	—	30	0.33 ± 0.2	3
8	139	300	—	22	0.17 ± 0.02	5.8
9	139	150	—	13	0.03 ± 0.01	6.4
10	692	150	75	18	0.25 ± 0.05	2.5
11	139	—	300	22	0.54 ± 0.23	4
12	139	—	150	12	0.12 ± 0.04	8

^a)^The amount of PDMS, ETAS and Ti-cat. used was 750 mg, 187 µL (213 mg) and 87 mg (50%, toluene), respectively.

^b)^Measured by placing film on a PCB and then adding 50 g circular cylindrical weight on top of the film.

#### Printing of the electrode for piezosensor

Composite GNPs/CB 300/150, 30 wt.% PDMS 139 k (1 g) (Table 1 Entry 2) diluted with toluene (9 mL) was blade coated on Neukasil PDMS substrate and used to investigate the performance as electrode for piezosensor. After cross-linking, samples with a diameter of 3 mm were cut, glued onto a safety frame, which was thereafter fixed in the setup used for long time measurement (see Supporting Information). The electrical resistance of electrodes was tested over 100’000 pressure cycles. The sample was regularly subjected to a contact force of 1.0 N at 1 Hz (0.5 seconds in contact and 0.5 waiting time), while a conductive PCB, connected to a 12 V (DC) voltage source using a resistor that limited the current to max. 100 mA, was used to measure the contact resistance.

## Results and Discussion

**Figure 1:**
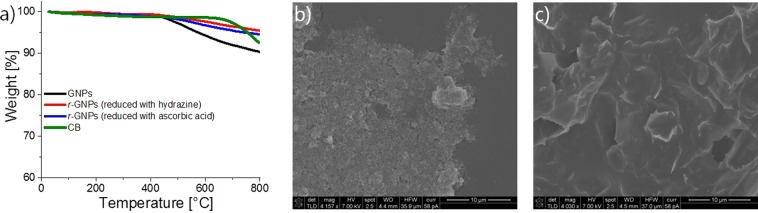
TGA of GNPs filler before and after reduction with hydrazine and ascorbic acid, respectively, and CB (**a**); SEM micrographs of CB (**b**) and GNPs (**c**).

To achieve lightweight and stretchable conductive composites two conductive fillers were used, namely GNPs and CB. GNPs were chosen because of their good electrical conductivity and the ability to improve the mechanical properties of the composites as a result of their large diameter of 25 µm. Since the bulk density of GNPs is in the range of 0.03–0.1 g/cm^3^, low amounts of GNPs can already fill a large volume and create conductive networks within the resulting composite material. It is known that the conductivity of GNPs can be increased via a reduction step in which functional groups which perturb the aromatic conjugation in graphene network are eliminated^36,37^. For this different reducing agents such as hydrazine and ascorbic acid can be used. However, in our case, the TGA investigations conducted on the reduced GNPs show a mass loss of only 5–6% (Fig. 1a). We concluded that the commercially acquired GNPs were already partially reduced, which is in accordance with previous reports, where a difference of 30 wt.% was measured after the reduction step^38^. Therefore, the composites were prepared entirely with the as received GNPs without further treatment. The second filler used, CB features good electric properties and tend to enhance the mechanical properties of the composites due to its large specific surface area attributed to the small sizes of the primary particles (~34 nm). Generally, the electrical conductivity is maximized if the CB exhibits large surface area, high number of primary particles per aggregate, thus leading to enhanced contacts between the adjacent particles^39–41^. This is why, CB of the grade Ketjenblack EC-600 JD from Akzonobel, which has a low bulk density of 0.1–0.12 g/cm^3^ was selected^17,42^. Similar to GNPs, CB also exhibited a high residual amount of material upon being subjected to thermal treatment (Fig. 1a). SEM micrographs of CB as well as of GNPs are illustrated in Fig. 1b,c.

**Figure 2:**
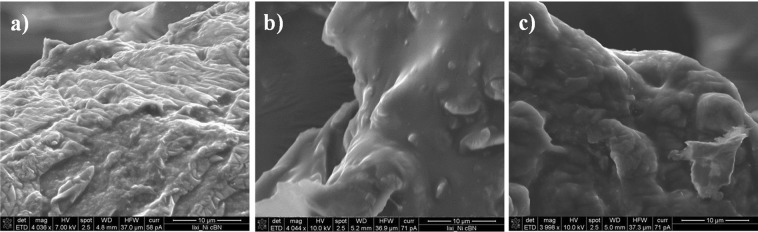
SEM micrographs of the cross-section of PDMS composites consisting of CB (**a**), GNPs (**b**), and a mixture of GNPs/CB (300/150, 30 wt.%) (**c**). The scale bar is 10 µm.

The production of homogenous nanocomposites with reproducible properties is quite challenging since it is essential to ensure a uniform dispersion of the filler within the polymer matrix. The composites were produced by mixing the components in a three-roll mill, where high shear forces act on agglomerates of the filler and break them apart, thus ensuring a homogenous dispersion of the filler within the matrix. The cross-linking of the composites in thin films can be achieved by different reaction conditions using a condensation reaction. It was, however, necessary to find the optimal conditions, since the presence of particles can inhibit the crosslinking of the PDMS chains^43,44^. Thus, the first preliminary tests were conducted using PDMS 139 k and GNPs. It should be mentioned that PDMS 139 k has silanol end-groups, which can be used for cross-linking with either (methylhydrosiloxane)–dimethylpolysiloxane, tetraethoxysilane, or ethyl triacetoxy silane^45^. When (25–35% methylhydrosiloxane)–dimethylpolysiloxane was used as cross-linker and Sn-cat, the cross-linking was not accomplished despite the long waiting time. The reason behind this may be the presence of some functional groups on the GNPs, which inactivates the Sn-cat. The reaction with ethyltriacetoxysilane (ETAS) cross-linker and Ti-cat was more reliable and the films cross-linked within few minutes after doctor blading and subsequent evaporation of the solvent at room temperature^46^. This cross-linking reaction proved to be compatible for both conductive fillers used and the synthesis of composite films was reproducible in terms of obtaining similar electrical properties for each batch of a composite with defined filler components. Adhesion tests performed with a strip of scotch tape clearly proved that the cross-linking reaction was successful, e.g., no residue of the composite was found on the scotch tape. Additionally, the reagents used are environmentally friendly and do not pose a hazard threat to the health. SEM micrographs conducted on the fractured films clearly show that the obtained composites are rather homogenous (Fig. 2).

The TGA of the PDMS matrices and the composites were performed under inert atmosphere in order to investigate the thermal stability (Fig. S3). In general, the tested materials start to degrade at around 450 °C. The titanium-catalyzed PDMS matrices exhibit a residual mass of around 10% at 800 °C. This can be attributed to the formation of TiO_2_ particles, which was confirmed by the opaque color of the pure PDMS film. Due to the presence of the fillers in the composites, the residual mass at 800 °C is higher compared to the neat PDMS matrices. Composites of GNPs with PDMS exhibit a higher temperature sensitivity and higher stability, thus rendering them attractive as conductive filler. The thermal stability of graphite nanocomposites is more enhanced compared to the composites where other carbonaceous fillers are applied^24^. This can be seen in this work, where the composites which include CB as filler degrade earlier than the composites where GNPs are used as single filler material only. Overall, the composites degrade faster with temperature compared to the PDMS matrix. This phenomenon could be attributed to the hindered cross-linking of some PDMS chains caused by the presence of the filler. Therefore, PDMS chains exhibit a higher freedom of movement in the composites due to the lack of covalent chemical bonding, which leads to their reduced thermal stability (See Supporting Information)^47^.

**Figure 3:**
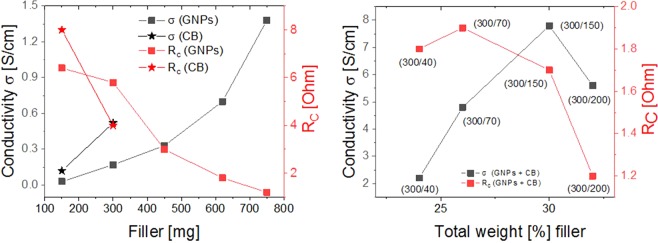
Development of conductivity *σ* and contact resistance (*R*_*c*_) of composites consisting of GNPs and CB (left) and development of conductivity and contact resistance of composites with a binary mixture of GNPs and CB at a ratio of (300/n), where n represents the amount of CB used (right).

After finding the optimum conditions for the formation of homogenous composites and for the cross-linking in thin films, the next step was to optimize their electrical conductivity. To achieve this we investigated the conductivity of composites with different content of GNPs, CB, and a mixture of the two. As expected, with increasing the content of GNPs from 13 wt.% to 42 wt.%, an almost linear increase in electrical conductivity from 0.03 to 1.4 S/cm was measured (Fig. 3, Table 1). A further increase of the GNPs content in the composites resulted in a dramatic deterioration of the mechanical properties of the composites and in inhibition of the cross-linking reaction due to the increased viscosity of the composite mixture^48^.

The CB/PDMS composites featured enhanced electrical conductivities compared to the GNPs/PDMS composites with the same filler loading. This is due to the larger contact area offered by CB particles, which have a small size. It has already been shown in earlier reports that higher electrical conductivities for PDMS composites were feasible with CB compared to graphite at the same weight concentration^48^. In our case, composites with a CB content of 12 and 22 wt.% exhibited conductivities of 0.12 and 0.54 S/cm, respectively. Similar conductivity values were previously reported for various CB fillers at the similar filler content but using different polymer matrices^23^. When the CB filler content was increased above 25 wt.%, the cross-linking reaction was inhibited and crack formation was observed in the films. Therefore, these films were not further investigated^49^.

To further enhance the electrical conductivity of the PDMS matrix, the composites with both fillers, i.e. binary mixtures of CB and GNPs, were prepared. For this, the amount of GNPs was kept constant at 300 mg, while the amount of CB was gradually increased from 40 to 200 mg, therefore limiting the content of CB in composites below 10 wt.% (Table 1). These hybrid filler composites exhibited superior electrical conductivity compared to the composites consisting of just one filler. A possible explanation to this improvement is the percolation due to formation of well-connected conductive pathways through the combination of the micron-sized graphite sheets and the nanosized CB^39,50^. The mixed fillers with different dimensions lead to a “bridging effect”, thus yielding conductivities exceeding the values of the composites for which only one filler component was used^51^. Eventually, these composites gave electrical conductivities ranging from 2.2 to 7.8 S/cm, with the highest conductivity value measured for a composite with a 300/150 mass ratio between GNPs and CB fillers (Table 1 Entry 2, Fig. 3). Previous reports have shown that a mass ratio of 300/150 between small/large particles in Vinnol resin is optimal to enhance the electrical conductivity^29,51^. The decrease in the electrical conductivity when the CB content was increased by changing the GNPs/CB mass ratio from (300/150) to (300/200) is attributed to the incorporation of air voids with increasing filler content and because of the lack of polymer chains to bind the fillers^29,50^.

The electrical properties of the conductive composites were further altered by using a PDMS of a higher molecular weight. For instance, by increasing the molecular weight of PDMS from 139 kg/mol to 692 kg/mol (Table 1, Entry 2b) a decrease in the electrical conductivity of the composite was noted^40^. The reason behind this may be the inhibited diffusion of the particles to create conducting networks during the cross-linking reaction. Additionally, the longer chains of the high molecular weight PDMS matrix will also be more effective in shielding the particles from each other, thus causing disruption of the conductive network.

The cross-linking reaction of the composites naturally occurs at room temperature since it only requires moisture from the air to hydrolyze ETAS followed by the condensation with the silanol end-goups of polysiloxane. In order to investigate the effect of the curing temperature on the electrical properties, the composites were dried at 80 °C under vacuum which is normally done with other PDMS cross-linking systems such as the crosslinking reaction between a hydrosilane and the silanol end groups of the polysiloxane. It was observed that the curing temperature of 80 °C did not heavily affect the sheet resistance of the conductive composites (Table 2). Furthermore, the filler contents of the conductive composites exceeds 50 vol% which is higher than the reported conductivity percolation threshold^52^. However, when the composites were post cured at 150 °C which is close to the T_g_ of PDMS, the electric conductivity/sheet resistance was altered. While the composite consisting of GNPs and CB (300/150, 30 wt.%) in PDMS 139 k exhibited an improvement in the electric conductivity after heating at 150 °C which led to an enhancement of 33%, the composite using GNPs (42 wt.%, PDMS 139 k) exhibited an enhancement of only 11%. In direct comparison, the electric conductivity of the composites using GNPs/CB (300/150, 30 and 18 wt.%) in PDMS 692 k featured a decrease of 32% and 4%, respectively (Fig. S4). In general, it can be concluded that the increase in the temperature until the T_g_ allowed the electrically conductive pathways to be fixated, and the particles are mobilized through the input of temperature as a result of reaching the T_g_ of the matrix^53^. Since GNPs are larger in size and less mobile than CB particles, the enhancement in the electric conductivities of the composite consisting only of GNPs was less substantial than for the composites utilizing the hybrid filler system due to the limited diffusion ability of the former. However, the enhancement in the electric properties was only observed in composites of PDMS 139 k. In this case, PDMS 692 k shielded off the particles much more efficiently which can be seen in the corresponding lower electric properties when using PDMS 692 k and therefore heating the composites to 150 °C did not lead to an enhancement in the electric properties. Instead, the PDMS 692 k composites exhibited a thermally-induced degradation of their electric properties after heating at 150 °C.

**Table 2:** The change in sheet resistance with curing temperature (GNPs, CB, GNPs/CB).

Entry from Table 1	PDMS *M*_w_ [kg∙mol^−1^]	GNPs [mg]	CB [mg]	^a)^Sheet resistance [Ω∙_ϒ_^−1^]	^b)^Sheet resistance [Ω∙_ϒ_^−1^]
2	139	300	150	8.6 ± 0.9	9.9 ± 2.4
2b	692	300	150	12.6 ± 2.4	12.3 ± 1.2
11	139	—	300	196 ± 93	217 ± 98
8	139	300	—	567 ± 64	529 ± 64

^a)^Cured at 25 °C for 24 h; ^b)^after heating at 80 °C under vacuum for 4 h.

**Figure 4:**
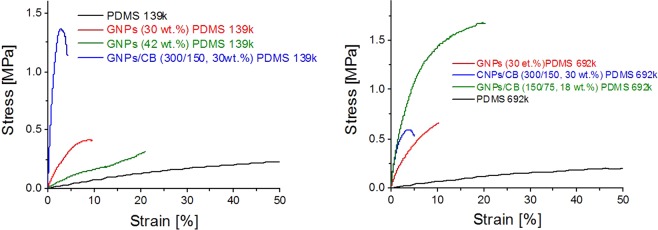
Tensile tests of GNPs/PDMS composites using PDMS 139 k (left) and PDMS 692 k as polymeric matrix (right).

Apart from the electrical properties, the mechanical properties are also important. Tensile measurements have shown that the pure PDMS matrix crosslinked with ETAS exhibited a Young’s modulus *Y*_*10%*_ = 0.73 MPa (PDMS 139 k) and *Y*_*10%*_ = 0.66 MPa (PDMS 692 k) and could be stretched up to 800% and 1250%, respectively (Fig. 4).

In comparison, the composites were less stretchable than the pure PDMS matrices. It is generally known that the incorporation of carbonaceous fillers leads to a deterioration of the mechanical properties as the materials become increasingly brittle. The composites consisting of GNPs (30 wt.%) exhibited a *Y*_*10%*_ = 5 MPa when dispersed in PDMS 139 k matrix. The use of PDMS 692 k led to an increase in the strain at break and less pronounced yielding of the GNPs/PDMS nanocomposites due to the lower crosslinking density. The use of PDMS 692 k led to an enhancement of the strain at break of the GNPs/PDMS composites from 9 to 13% although a slight increase in *Y* from 5.4 to 6.2 MPa, results that are in agreement with the literature^54^. An increase in the GNPs content in PDMS 139 k from 30 to 42 wt.% led to a decrease in *Y*_*10%*_ from 5.4 to 1.5 MPa, which could be attributed to increasing agglomeration of the filler within the matrix and the dewetting of the polymer at the interphase^55,56^. Composites with high CB content were rather brittle, therefore limiting the potential of CB^51^. Because of this, no mechanical tensile tests were conducted with these composites.

The incorporation of CB as second filler can improve the mechanical properties of composites. For example Acosta *et al*. observed that the introduction of Fe allowed the dispersion of CB at a content of ≥40 wt.%, which facilitated the creation of conductive networks and improved electrical properties^49^. In comparison to the composites consisting of GNPs, the binary GNPs/CB mixture (300/150) at the same wt.% filler (30 wt.%) could only be stretched at lower strains (≤5%) prior to rupture (Fig. 4). The presence of CB in the material led to an enhancement in *Y*, which is attributed to the small size of the CB particles which reinforce the composite^56,57^. Therefore, the composites consisting purely of GNPs exhibit superior flexibility than the composites consisting of the binary filler mixture. The composite with GNPs/CB ratio of 300/150 (30 wt.%) in PDMS 139 k matrix exhibited a *Y* of 51 MPa. The increase in the PDMS molecular weight reduced the *Y*_*10%*_ of the material from 51 to 12.1 MPa, although an enhancement in the strain-at-break was hardly noticed. If the GNPs/CB ratio was kept constant at 2:1 and the total amount of filler was reduced by 50% (GNPs/CB at 150/75, 18 wt.%), the strain-at-break increased to almost 20% strain and the *Y* decreased to 11.2 MPa. As mentioned earlier, this comes at the expense of the electrical conductivity, as the reduction in the filler content also leads to a decline in the electrical properties of the materials (Table 1).

**Figure 5:**
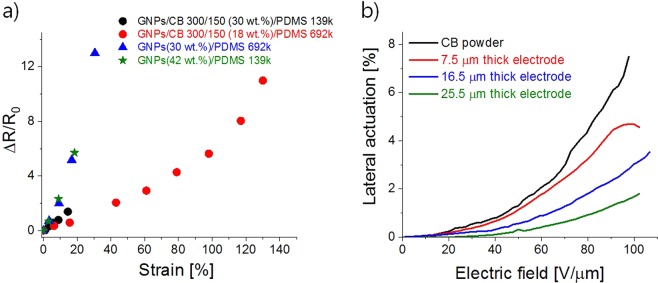
Strain-dependent relative change of electrical resistance in the GNPs/PDMS and GNPs/CB/PDMS nanocomposites (**a**) and lateral actuation strain as function of electric field for actuators constructed with Elastosil dielectric film and having electrodes of GNPs/CB 300/150 (18 wt.%) PDMS 692 k with a thickness of 7.5 μm, 16.5 μm, and 25.5 μm as well as of an actuator for which CB powder was used (**b**).

Although Phillips *et al*. reported graphite/CB/polymer nanocomposites with high conductivities, the effect of the mechanical strain on their electrical properties was neglected^29^. Hence, the composites prepared in this work were subjected to strain-dependent resistance measurements. Despite the increased stiffness due to the presence of the filler, the use of PDMS as polymer matrix provides flexibility to the composites. It is well known that composite containing small sized fillers exhibits a higher relative change of electrical resistance ∆R/R_0_ than the composite containing filler that has a high aspect ratio. The conductive network of the former can accommodate higher deformation because the particles can slip away from each other when the material is strained^23,51^. Therefore, composites consisting solely of CB would exhibit a higher increase of ∆R/R_0_ at similar strains compared to composites with graphene or graphite. The composites consisting of only GNPs filler exhibited a sharp increase in ∆R/R_0_ (Fig. 5)_._ A composite of GNPs and PDMS 139 k with a filler content of 42 wt.% showed an increase of ∆R/R_0_ of 5.7 prior to the rupture of the conductive composite at a strain of 18.5%. When the amount of filler was reduced to 30 wt.% and PDMS 692 k was used, a ∆R/R_0_ of 13 was measured at 30.5% strain, where the material ruptured. The slope of ∆R/R_0_ seemed identical for these two composites, which indicates a similar response of the conductive network created by the filler on the imposed deformation.

In direct contrast, the use of hybrid fillers consisting of CB and GNPs would lead to materials with lower ∆R/R_0_ due to the densification of the conductive network as the nanosized CB fills the voids between the GNPs sheets within the material, thus enhancing the contact between the filler materials. As a result, the conductive network is more robust with respect to the changes in electrical resistance with strain, as shown in Fig. 5a. Here, the GNPs composites feature a sharper increase in the ∆R/R_0_ compared to the hybrid filler composites with a GNPs/CB mass ratio of (300/150, 30 wt.%). With filler contents of 30 wt.% or higher, the conductive network barely survives 20% strains. The reduction of the filler content and the increase in the molecular weight *M*_n_ of the PDMS leads to conductive composite materials, which feature a conductive network even at high strains. For instance, the material consisting of GNPs/CB (150/75, 18 wt.%) in PDMS with *M*_n_ = 692 kg/mol features a linear response of ∆R/R_0_ up to 100% uniaxial strain, which surpasses the flexibility and linear response region of previously reported carbonaceous electrode materials^20,58^, and is comparable to CNFs/PDMS composites previously reported and suitable as large strain-sensor materials^19^. Overall, compared to previously reported materials^23,58^, the electrodes exhibit sufficient conductivity to perform as electrodes for sensor applications. While those electrodes exhibit conductivities between 10^−6^ to 10^−2^ S/cm, all the reported electrodes here feature electric conductivities between 0.25–7.8 S/cm, and they can be strained up to 100% by choosing the filler content and composition as well as the properties of the resulting PDMS matrix such as the composite consisting of GNPs/CB (150/75, 18 wt.%) in PDMS with *M*_n_ = 692 kg/mol. Furthermore, this composite exhibits superior ∆R/R_0_ values to recently reported CB/PDMS and CNTs/PDMS composites, respectively, at similar mechanical strains, and both have been demonstrated to have potential applications as strain sensors^52^.

To demonstrate the feasibility of the developed material as electrode for DET, the GNP/CB 300/150 (18 wt.%) composite in PDMS 692 k was selected because it showed the smallest change in ∆R/R_0_ with strain. Actuators having electrodes with three different thicknesses and a diameter of 1 cm, were screen printed on Elastosil films (50 μm). The lateral actuation strain at different electric fields for actuators with different electrode thicknesses is compared to the one where CB powder electrodes are used. As can be seen, the thickness of the electrode strongly influences the actuation properties, e.g. the thicker the electrode, the lower the actuation strain. This is not unexpected, since the elastic modulus of the composite used is higher as compared to the elastic modulus of Elastosil film. It should be noted that the actuation strain of CB electrode and of the 7.5 μm thick electrode up to an electric field of 70 V/μm almost overlap. Therefore, this electrode thickness is considered attractive for actuator application. The actuation strain at 70 V/μm decreased from 2.5% for a 7.5 μm thick electrode to 0.6% for a 25.5 μm thick electrode, respectively. Here, only one actuator with a thickness of 25.5 μm could be prepared and measured. While the later electrode thickness is unattractive for actuator application, where large actuations are desirable at lowest electric field, this electrode thickness could be suitable for DETs operated in the generator mode (DEG). In DEG, it is important to have a reduced actuation, because the amount of energy harvested can be increased^2^.

**Figure 6:**
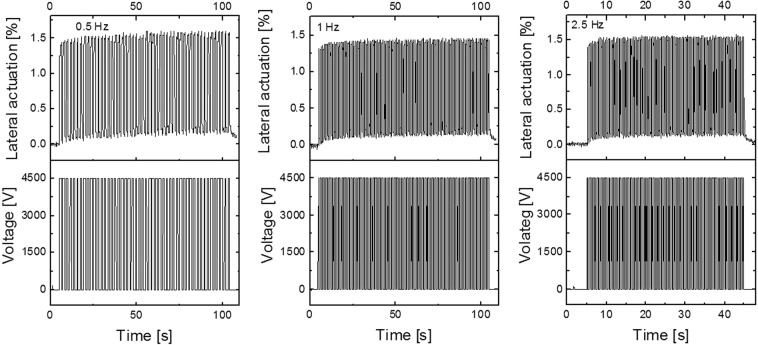
Cyclic lateral actuation strain at 4500 V at 0.5 Hz (50 cycles), 1 Hz (100 cycles), and 2.5 Hz (100 cycles) for an actuator having an electrode with a thickness of 25.5 μm. The actuators were 7.5% prestrained.

Cyclic actuation tests conducted on devices that have electrodes with a thickness of 25.5 μm show negligible hysteresis at the investigated frequency range of 0.5 Hz, 1 Hz, and 2.5 Hz, which are relevant for transducers applications (Fig. 6). However, actuators constructed with thinner electrodes (7.5 μm) show a slightly lower actuation strain when increasing the frequency from 0.5 Hz to 10 Hz, which is likely related to the conductivity of the electrodes (Figs S5–S9).

**Figure 7:**
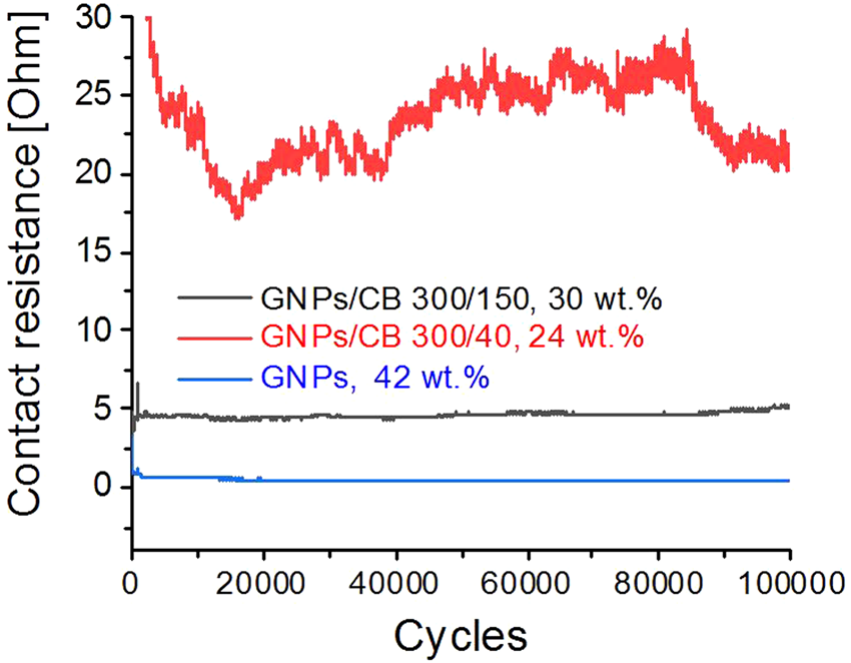
Life-cycle tests of electrode materials consisting of GNPs (42 wt.%), GNPs/CB 300/150, 30 wt.% and GNPs/CB 300/40, 24 wt.%.

Lastly, the performance of the developed materials as electrode for pressure sensors was evaluated. Initially, the composites of GNPs with PDMS 139 k were subjected to life-cycle tests, where the electrical resistance of electrodes was tested over 100’000 pressure cycles. Compared to the contact resistance values listed in Table 1, it has to be mentioned that the samples used for the lifecycle tests were pills with a diameter of 3 mm. These pills were subjected to a contact force of 1.0 N (equivalent to about 102 grams acting on them). When composites with 30 wt.% GNPs was tested, the electrical resistance was so large that it was concluded that this material was not suitable to be used as electrode for piezosensors. When the amount of GNPs was increased to 42 wt.%, the electrical resistance was below 1 Ω which is a strong argument for the use of this material as electrode material for pressure sensors. Apart from the larger resistance during the first few cycles, the electrical resistance stabilized itself and, more importantly, the electrode was able to sustain 100’000 pressure cycles, while maintaining its electric properties. In comparison, the composites using binary filler components of GNPs and CB (300/150, 30 wt.% and 300/40, 24 wt.%), exhibited lower contact resistance compared to the 42 wt.% GNPs in PDMS 139 k (Fig. 7). The electric resistance of the composites during the life cycle tests was determined at around 5 and 25 Ω, respectively, but similar to the GNPs/PDMS composite (42 wt.%), they can also survive 100’000 cycle tests. Overall, industrial applications require materials exhibiting resistance values below 10 Ω and a life-time surpassing 100’000 pressure cycles, therefore the GNPs/PDMS (42 wt.%) and GNPs/CB (30/150, 30 wt.%)/PDMS composites can be used as potential electrodes for piezosensor applications.

The application potential of some of the developed electrode materials is high. Not only are the materials used accessible, cheap and environmental friendly, but they can be processed into thin films by screen printing, a process that is used by the industry for large scale production.

## Conclusions

Conductive composites consisting of GNPs and CB in two different PDMS matrices were developed. They can be processed into thin films by screen printing as well as by blade coating. A composite GNPs/CB (150/75, 18 wt.%) shows a moderate change of ∆R/R_0_ with strain, which was found to be very attractive for DETs as was observed from the actuator testing. It was shown that this composite functions well as electrode in actuators. Additionally, just by adjusting the electrode thickness, the same electrode material may function well in actuators or in generators. The best material in terms of conductivity was achieved with a PDMS composite consisting of GNPs/CB (300/150, 30 wt.%). It showed a rather high conductivity of 7.8 S/cm and a contact resistance of 5 Ω and was found to be very attractive as electrode for piezosensors. Similarly, the composite consisting only of GNPs (42 wt.%) exhibited a lower electric conductivity, but a contact resistance of 0.5 Ω. Both composites showed almost constant electrical properties over 100’000 press cycles. Because the developed electrodes can be easily applied on different dielectric materials by screen printing, a process that can be used on large scale production of different devices, and because they are commercially available, cheap, lightweight, and highly conducting, their application in commercial devices can be envisioned.

## Supplementary information

Supporting information

## Data Availability

The datasets generated during and/or analysed during the current study are available from the corresponding author on reasonable request.
