# Supplementary material for: Conductive silicone elastomers electrodes processable by screen printing
Source: Sci Rep. 2019 Sep 16;9:13331. doi: 10.1038/s41598-019-49939-8 (PMC6746820; doi:10.1038/s41598-019-49939-8)
Supplement: Supplementary file 1 — Supporting information [file 41598_2019_49939_MOESM1_ESM.docx]

**Supporting information**

**Conductive silicone elastomers electrodes processable by screen printing**

Jose Enrico Q. Quinsaat,^1^* Iurii Burda,^2^ Ronny Krämer,^3^ Daniel Häfliger,^3^ Frank A. Nüesch,^1,4^ Mihaela Dascalu,^1^ Dorina M. Opris^1^*

Dr. J. E. Q. Quinsaat, Prof. F. A. Nüesch, Dr. M. Dascalu, Dr. D. M. Opris

^1^Swiss Federal Laboratories for Materials Science and Technology Empa, Laboratory for Functional Polymers, Ueberlandstr. 129, CH-8600, Dübendorf, Switzerland

E-mail: dorina.opris@empa.ch

I. Burda

^2^Swiss Federal Laboratories for Materials Science and Technology Empa, Laboratory for Mechanical Systems Engineering, Ueberlandstr. 129, CH-8600, Dübendorf, Switzerland

R. Krämer, Dr. D. Häfliger

^3^Sateco AG, Tumigerstr. 111, CH-8606, Naenikon-Uster, Switzerland

Prof. F. A. Nüesch

^4^Ecole Polytechnique Fédérale de Lausanne (EPFL), Institut des Matériaux, Station 12, CH 1015, Lausanne, Switzerland


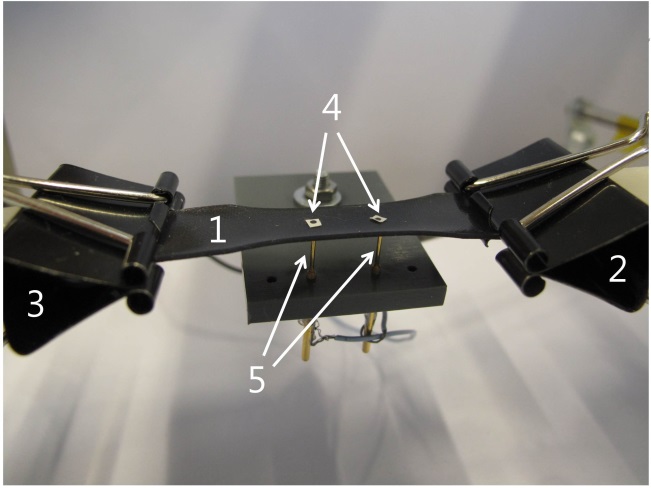


**Fig. S1.** Experimental setup for the electromechanical characterization of the PDMS composites: 1 – dumb-bell sample; 2, 3 – clamps; 4 – markers for strain measurement; 5 – voltage measurement pins.

**Fig. S2.** Photograph of a conductive layer of a GNPs/CB 300/150, 30wt.% PDMS 138k composite coated on a transparent silicone film and insert two round samples (*Φ* = 3 mm) cut to be used for the resistance measurement (a). Photographs of PCb on which the safety frame (blue) with the round sample glued in the middle (turned up - top and turned down -bottom) (b). Photo of the PCB used with contact width of 0.5 mm and a distance between contact paths of 0.3 mm (c). The setup used for long time measurement of the contact resistance (d) and (e).

**Fig. S3.** The TGA of the PDMS matrices and of the prepared composites.

**Fig. S4.** The change in the electrical conductivity after heating selected composites at 150 °C for 24h and cooling back to room temperature. Positive values mean an enhancement in the conductivity, while negative values mean a decrease in the conductivity.

**Fig. S5.** Cyclic lateral actuation strain at 4000 V at 0.5 Hz (100 cycles) for an actuator having an electrode with a thickness of 7.5 μm. The actuators were 7.5 % prestrained.

**Fig. S6.** Cyclic lateral actuation strain at 4000 V at 1 Hz (100 cycles) for an actuator having an electrode with a thickness of 7.5 μm. The actuators were 7.5 % prestrained.

**Fig. S7.** Cyclic lateral actuation strain at 4000 V at 2.5 Hz (100 cycles) for an actuator having an electrode with a thickness of 7.5 μm. The actuators were 7.5 % prestrained.

**Fig. S8.** Cyclic lateral actuation strain at 4000 V at 5 Hz (100 cycles) for an actuator having an electrode with a thickness of 7.5 μm. The actuators were 7.5 % prestrained.

**Fig. S9.** Cyclic lateral actuation strain at 4000 V at 10 Hz (100 cycles) for an actuator having an electrode with a thickness of 7.5 μm. The actuators were 7.5 % prestrained.
